# Measurement of Low Carbon Economy Efficiency with a Three-Stage Data Envelopment Analysis: A Comparison of the Largest Twenty CO_2_ Emitting Countries

**DOI:** 10.3390/ijerph13111116

**Published:** 2016-11-09

**Authors:** Xiang Liu, Jia Liu

**Affiliations:** 1 Economics and Management School, Wuhan University, Wuhan 430072, China; xiangliu1018@gmail.com; 2 School of Information and Safety Engineering, Zhongnan University of Economics and Law, Wuhan 430073, China

**Keywords:** efficiency, low carbon economy, data envelopment analysis, stochastic frontier approach, three-stage DEA

## Abstract

This paper employs a three-stage approach to estimate low carbon economy efficiency in the largest twenty CO_2_ emitting countries from 2000 to 2012. The approach includes the following three stages: (1) use of a data envelopment analysis (DEA) model with undesirable output to estimate the low carbon economy efficiency and calculate the input and output slacks; (2) use of a stochastic frontier approach to eliminate the impacts of external environment variables on these slacks; (3) re-estimation of the efficiency with adjusted inputs and outputs to reflect the capacity of the government to develop a low carbon economy. The results indicate that the low carbon economy efficiency performances in these countries had worsened during the studied period. The performances in the third stage are larger than that in the first stage. Moreover, in general, low carbon economy efficiency in Annex I countries of the United Nations Framework Convention on Climate Change (UNFCCC) is better than that in Non-Annex I countries. However, the gap of the average efficiency score between Annex I and Non-Annex I countries in the first stage is smaller than that in the third stage. It implies that the external environment variables show greater influence on Non-Annex I countries than that on Annex I countries. These external environment variables should be taken into account in the transnational negotiation of the responsibility of promoting CO_2_ reductions. Most importantly, the developed countries (mostly in Annex I) should help the developing countries (mostly in Non-Annex I) to reduce carbon emission by opening or expanding the trade, such as encouraging the import and export of the energy-saving and sharing emission reduction technology.

## 1. Introduction

Climate change represents a growing threat to the sustainable development of human beings. The continuing CO_2_ emissions caused by economic activities is recognized as one of the major contributors to climate change [1]. In order to cope with it, the United Nations organized the countries all over the world to attend the United Nations Framework Convention on Climate Change (UNFCCC) to negotiate about fighting against global warming. Moreover, low carbon economy is introduced as a new economic development mode aiming at promoting carbon reductions without hampering economic growth [2].

Since improving efficiency and productivity is considered as one of the most crucial approaches to promoting economic development [3], a growing number of studies have indicated the performance and quality of the economic development by evaluating and analyzing economic efficiency. In the field of low carbon economy development, “low carbon economy efficiency” can be viewed as a kind of environmental efficiency which indicates the capacity of using fewer input resources to increase the Gross Domestic Product (GDP), while producing fewer carbon emissions. Therefore, low carbon economy efficiency reflects the performance of developing a low carbon economy.

In recent literature, data envelopment analysis (DEA) has been widely employed to evaluate the economic efficiency and environmental efficiency by various models, including modified value-chains DEA model [4], Range-Adjusted Measure DEA approach [5], and super-efficiency DEA window analysis [6]. However, traditional DEA models that refer to “constant inputs producing more outputs” or “fewer inputs producing constant outputs” may not evaluate low carbon economy efficiency appropriately because CO_2_ emissions are not the traditional output (such as GDP) in DEA models. Specifically, GDP is a desirable output recognized as “the more the better”, while CO_2_ emissions is an undesirable output recognized as “the less the better”. In recent years, many researchers proposed various methods evaluating the efficiency of Decision Making Units (DMUs) with undesirable outputs in DEA models. Ramanathan et al., considered the reciprocal values of CO_2_ emissions as outputs in traditional DEA model to investigate the linkages among CO_2_ emissions, GDP growth and energy consumption [7]. Tyteca et al. directly considered undesirable output as input in standard DEA models [8]. However, these transformations of CO_2_ emission values may not reflect the actual production process that the undesirable and desirable output are usually produced simultaneously. Additionally, it cannot influence DEA results when used as input [9].

To reasonably evaluate the efficiency with DEA model that considers desirable output (GDP) and undesirable output (CO_2_ emissions) simultaneously, current papers have proposed several DEA models based on environmental DEA technology, and the reviews of these models and their applications are presented by Zhou et al. [10], Liu et al. [11] and Song et al. [12]. Among these different methods, the slack-based measure (SBM) model proposed by Tone [13] is the most widely used one [14]. Following the idea of Tone [13], Zhou et al. [15] proposed a slacks-based DEA model, namely SBM-DEA mode, in which more desirable outputs, less undesirable outputs and less inputs are recognized as “efficient”. Therefore, the SBM-DEA model provides a higher discriminating power in modeling environmental performance [15]. Due to the advantage of SBM-DEA model, some literature has utilized this model to study environmental efficiency. Zhou et al. took CO_2_ emissions as an undesirable output and GDP as a desirable output in the SBM-DEA model to estimate the performances of CO_2_ emissions of those countries members of the Organization for Economic Co-operation and Development (OECD) from 1998 to 2002 [15]. Hu and Kao saw the input slacks obtained from the DEA results as the target of the total reducing amount and used this model to measure energy-saving target ratios for 17 economies of the Asia-Pacific Economic Cooperation (APEC) from 1991 to 2000 [16]. Choi et al. employed SBM-DEA model to evaluate the CO_2_ emission and energy efficiencies of 30 provinces in China [14]. Li et al. extended the SBM-DEA model to Super-SBM model to measure regional environmental efficiency in China from 1991 to 2010 [17]. In addition, the SBM-DEA model was also used to evaluate the efficiency of cities [18], industrial sectors [19] and banks [20]. The advantage of using the SBM model to evaluate low carbon economy efficiency is elaborated by Zhou et al. [15] and Choi et al. [14].

However, one weakness of using the SBM-DEA model to compare the different environmental efficiency performances among different regions is that the influences of external environmental variables on slacks are ignored. These external environmental variables show great differences among areas and contribute to the slacks of inputs and cause the bias of actual efficiency performance [21]. To address this issue, Fried and Lovell [22] proposed a three-stage DEA model which includes the following three stages: (1) utilization of traditional methods to calculate the slacks of inputs; (2) use of regression models to estimate the relations between the input slacks and external environmental variables to eliminates the impacts of them on inputs; (3) re-estimation of the net efficiency by the adjusted inputs which eliminates the impact of external environmental variables [23]. The three-stage DEA model is better than traditional DEA models because it uses individual environmental effects and statistical white noise which vary among different DMUs to adjust the inputs [24].

Li and Lin [25] employed a three-stage DEA model to evaluate the green productivity growth of China’s manufacturing sector. Their research takes China’s energy-saving policies and measures as external environmental variables. They pointed out that the green production efficiency of China’s manufacturing industry would decrease while implementing energy-saving policies and measures, which means that there is still a long way to go in energy saving policies and measures of China. The three-stage DEA model is also employed by Bi et al. to propose that environmental regulation has a significant impact on China’s thermal power generation and decreasing the discharge of major pollutants can improve both energy performance and environmental efficiency [26]. Based on the three-stage DEA model, Yang and Pollitt evaluated the performance of the Chinese coal-fired power plants and Chen et al. studied the energy efficiency of China’s construction industry [27,28].

With the increase of international focus on reducing carbon emissions and developing low carbon economy issue, it is of necessity to integrate the external environmental factors into the study of low carbon economy efficiency. This paper employs a three-stage DEA model with undesirable outputs to calculate the efficiency of the largest 20 CO_2_ emitting countries. There are two innovative points of it. Firstly, by using the three-stage DEA model with undesirable outputs to measure low carbon economy efficiency of different countries, the influence of external environmental factors on efficiency can be eliminated, which means that the values of the efficiencies can be thought of the government management capacity to promote low carbon economy development. Secondly, analyzing and comparing the performance of low carbon economy efficiency of the top 20 countries in controlling carbon emissions which account for 80.5% of the amount of global CO_2_ emission provides helpful and meaningful guidelines for most countries to implement appropriate policies to develop a low carbon economy.

This study is structured as follows: the method of the three-stage DEA model with undesirable outputs and data used in this paper are briefly introduced in Section 2. The performances of low carbon economy efficiency (hereinafter called “Efficiency”) in different countries during the period from 2000 to 2012 are discussed in Section 3. The final section presents the conclusions and some policy implications.

## 2. Materials and Methods

### 2.1. Three-Stage Undesirable Output SBM-DEA Model

The first stage of this model is using SBM DEA model with original input and output variables to calculate the total input slacks of DMUs and Efficiency without considering external environmental variables.

Based on Tone [13] and Zhou et al. [15], the undesirable output SBM-DEA model can be defined as follows: given that there are *n* DMUs in production system *P*, and each DMU has *m* inputs, *S*_1_ desirable outputs and *S*_2_ undesirable outputs. $X=\left[x_{1},x_{2},\ldots ,\;x_{n}\right]\in R^{m\times n}$, $Y^{g}=\left[y_{1}^{g},y_{2}^{g},\;\ldots ,\;y_{n}^{g}\right]\in R^{S_{1}\times n}$, and $Y^{b}=\left[y_{1}^{b},y_{2}^{b},\;\ldots ,\;y_{n}^{b}\right]\in R^{S_{2}\times n}$ represent inputs, desirable outputs, and undesirable outputs, respectively. In this paper, we assume X>0, $Y^{g}>0$, and $Y^{b}>0$.

The production possibility set (*P*) is defined as:

(1) $$P=\{\left(x,y^{g},y^{b}\right)|x\geq X\lambda ,\;y^{g}\leq Y^{g}\lambda ,y^{b}\geq Y^{b}\lambda ,\;\sum \lambda =1\}$$

where $\lambda \in R^{n}$ is the intensity vector, and ∑​λ=1 represents the assumption of variable returns to scale (VRS).

DMU_0_ $\left(x_{0},y_{0}^{g},y_{0}^{b}\right)$ is defined as the efficient if there is no vector $\left(x,y^{g},y^{b}\right)\in P$ that simultaneously satisfies $x_{0}\geq x,y_{0}^{g}\leq y^{g}\;and\;y_{0}^{b}\geq y^{b}$.

Therefore, the SBM-DEA model with undesirable outputs can be written as:

(2) $$\rho =\min\frac{1-\frac{1}{m}\sum_{i=1}^{m}\frac{s_{i}^{-}}{x_{i0}}}{1+\frac{1}{s_{1}+s_{2}}\left(\sum_{r=1}^{s_{1}}\frac{s_{r}^{g}}{y_{r0}^{g}}+\sum_{r=1}^{s_{2}}\frac{s_{r}^{b}}{y_{r0}^{b}}\right)}\;subject\;to\;\{\begin{array}{c} x_{0}-X\lambda +S^{-}=0\\ y_{0}^{g}-Y^{g}\lambda +S^{g}=0\\ y_{0}^{b}-Y^{b}\lambda -S^{b}=0\\ S^{-}\geq 0,S^{g}\geq 0,\;S^{b}\geq 0,\lambda \geq 0 \end{array}$$

where $s^{-},\;s^{g},\;s^{b}$ correspond to slack variables of inputs, desirable outputs, and undesirable outputs, respectively. The objective function strictly decreases with respect to $s_{i}^{-}\left(\forall i\right),\;s_{r}^{g}\left(\forall r\right),\;s_{r}^{b}\left(\forall r\right)$ and the objective value satisfies 0<ρ≤1. Let an optimal solution of the above program be $\left(\lambda^{*},s^{-*},s^{g^{*}},s^{b^{*}}\right)$, and if and only if $\rho =1,\;s^{-*}=0,\;s^{g^{*}}=0,\;s^{b^{*}}=0$, the DMU is efficient.

In the second stage, the stochastic frontier approach (SFA) is employed to adjust the input slack variables. Based on Aigner et al. [29] and Meeusen et al. [30], the SFA regression model taking the inputs slacks as dependent variables and the external environmental variables as independent variables can be expressed as:

(3) $$s_{ij}=f^{i}\left(z_{j};\;\beta_{i}\right)+v_{ij}+\mu_{ij}$$

in which $s_{ij}$ is the slack of *j*^th^ DMU (different countries in this paper) in *i*th input which refers to $s^{-},\;s^{g},\;and\;s^{b}$ in SBM-DEA model; $f^{i}\left(Z_{j};\beta_{i}\right)$ is the deterministic feasible slack frontier; $z_{j}=\left(z_{1j},z_{2j},\ldots ,\;z_{Kj}\right)$ is environmental variables, where K is the number of environmental variables; $\beta_{i}$ is the environmental factor parameter vector for estimation; $v_{ij}$ and $\mu_{ij}$ are independent variable, and $v_{ij}+\mu_{ij}$ is the error term; $v_{ij}\sim iidN\left(0,\;\sigma_{v}^{2}\right)$ is statistical noise; and $\mu_{ij}\sim iidN^{+}\left(\mu^{i},\sigma_{\mu }^{2}\right)$ refers to the managerial inefficiencies.

The estimator of $\mu_{ij}$ can be obtained by using the equation provided by Kumbhakar and Lovell [31]:

(4) $$\hat{E}\left(\mu_{ij}|\mu_{ij}+v_{ij}\right)=\sigma_{*}\left[\frac{\phi \left(\frac{\varepsilon_{i}\lambda }{\sigma }\right)}{\Phi \left(\frac{\varepsilon_{i}\lambda }{\sigma }\right)}+\frac{\varepsilon_{i}\lambda }{\sigma }\right]$$

where $\sigma_{*}^{2}=\sigma_{\mu }^{2}\sigma_{v}^{2}/\sigma^{2}$, $\varepsilon_{i}=\mu_{ij}+v_{ij}$, $\sigma^{2}=\sigma_{\mu }^{2}+\sigma_{v}^{2}$. ϕ(⋅), Φ(⋅) are density function and distribution function of standard normal distribution, respectively. Therefore, the estimator of $v_{ij}$ can be calculated by:

(5) $$E\left[v_{ij}|\mu_{ij}+v_{ij}\right]=s_{ij}^{-}-z_{j}\hat{\beta_{j}}-\hat{E}[\mu_{ij}|\mu_{ij}+v_{ij}]$$

As such, the adjustment equation can be written as:

(6) $$x_{ij}^{A}=x_{ij}+\left[max_{j}\left\{z_{j}\hat{\beta_{i}^{-}}\right\}-z_{j}\hat{\beta_{i}^{-}}\right]+\left[max_{j}\left\{\hat{v_{ij}^{-}}\right\}-\hat{v_{ij}^{-}}\right]\;i=1,2,\ldots ,m;j=1,2,\ldots ,n$$

where $x_{ij}^{A}$ is the adjusted value of $x_{ij}$ which is the *i*^th^ original input of *j*^th^ DMU. The first square bracket of Equation (6) represents the adjustment of all DMUs in the same operation environment status, while the second square bracket is for the adjustment of the statistical noise of all DMUs in the same condition. When the environmental factors and statistical noise are the same, managerial inefficiencies constitute the last factor. According to the SFA results, the input variables of each DMUs are adjusted to the same environmental conditions and statistical noise.

In the third stage, we re-evaluate Efficiency by using the adjusted input and output obtained from the second stage with the Undesirable output SBM-DEA model proposed in the first stage. In other words, the models in stage 1 and stage 3 are based on the two separate samples. Since the environmental factors and statistical noise have been eliminated in the second stage, the results of Efficiency calculated in the third stage are a pure managerial factor that bears a more realistic reflection of managerial efficiency.

### 2.2. Data and Variables

The empirical study covers the largest 20 CO_2_ emitting countries in the world in the period from 2000 to 2012. The amount of CO_2_ emissions of these countries is 27.67 billion tonnes (metric ton) which accounts for 80.5% of the total CO_2_ emissions of the world in 2012. Ten of these twenty countries are Annex I countries of the UNFCCC, these industrialized (developed) and “economies in transition” (EITs) countries have the obligations to decrease their emissions of greenhouse gases. The other ten countries are involved in the Non-Annex I of the UNFCCC which are encouraged to promote carbon emissions.

#### 2.2.1. Inputs and Outputs

The purpose of developing low carbon economy is simultaneously promoting GDP growth and decreasing greenhouse gases emissions. Therefore, in the undesirable output SBM-DEA model, the amount of greenhouse gases emissions and GDP are taken as undesirable output and desirable output, respectively. Of the six kinds of greenhouse gases emissions, CO_2_ attracts major attention at present. Given its importance and the availability of data, CO_2_ emissions are selected as the respective indicator of greenhouse gases in this research. The amount of energy consumption (E), labor force (L), and capital stock (K) are taken as inputs in the model. The values of CO_2_ emissions by country from 2000 to 2012 are acquired from “BP statistical review of world energy 2013” [32]. The capital stock is calculated by perpetual inventory approach based on the gross fixed capital formation provided by the World Bank. The detailed calculation process is elaborated by Chen [33]. All data of other inputs and outputs are obtained from “World Bank Open Data” [34]. Moreover, all price values are converted to those in 2000 according to GDP deflator. Descriptive statistics of the inputs and outputs are presented in Table 1.

#### 2.2.2. External Environmental Factors Impact Efficiency

Since the performance of Efficiency is affected by other socio-economic factors which vary from country to country, several external environmental factors are taken into account for the purpose of eliminating the influences of these environmental variables on Efficiency in the second stage of the three-stage SBM-DEA model. This research focuses on five important variables to limit possibilities, in spite that there are much more determinants influencing low carbon economy development. In the economic aspect, the country which can provide enough financial support for the government to manage environment issues such as developing low carbon economy usually has a developed economy [17]. Consequently, GDP per capita is taken to measure the relative economic scale of a country. Besides, the impacts of industry structure on CO_2_ emissions is quite different in previous studies [35,36]. It is clear that China would experience an accelerated growth in the amount of energy consumption and CO_2_ emissions during the process of heavy industrialization. The ratio of industrial value added to GDP is taken as an indicator for measuring industry structure. Urbanization shows a positive contribution to energy-related carbon emissions growth due to increased building and infrastructure construction, as well as higher residential energy consumption and surging electricity demand [37].

The urbanization rate in China has greatly increased from 36.2% in 2000 to 52.6% in 2012, which makes carbon emission reduction very difficult and urgent [38]. In this paper, the proportion of urban residents in the total population represents the urbanization rate. Moreover, carbon emission is a typical public issue which government should pay much attention to. The China’s investment-driven growth model implies that the national government’s attitude towards the environmental problems can be seen from the amount of government financial investment. Moreover, the proportion of financial expenditure in GDP is considered as a proxy indicator of government support. Last but not the least, the ongoing expanding of imports and exports is a significant driving force for GDP growth and CO_2_ emissions in China [39] due to the introduction of advanced technology, equipment, and management experience. However, CO_2_ emissions embedded in products can promote the amount of CO_2_ emissions. It is estimated that CO_2_ emissions embedded in China’s domestic production for exports accounted for 19% of the total emissions in 2008 [40]. Therefore, international trade is of great importance in CO_2_ emission. The ratio of the amount of imports and exports to GDP is used as a proxy indicator. Table 2 shows the details of these indicators.

## 3. Results and Discussion

### 3.1. Stage I: Efficiency Based on Traditional Undesirable Output DEA Model

The scores of Efficiency in various countries are calculated by the traditional undesirable output DEA model which is relative to Equation (2) based on the raw data of the inputs and outputs. The results can be found in Table 3.

Table 3 illustrates that nine countries were technically efficient in the periods 2000–2012 as the corresponding Efficiency scores are “1”. This indicates that these ten countries show more balanced development between GDP growth and CO_2_ emissions by using the same resources (inputs) than the others. Among these nine efficient countries, six are in Annex I in UNFCCC (United States, Japan, United Kingdom, Italy, Australia, France) and three are Non-Annex I ones (Saudi Arabia, South Africa, Thailand).

The average Efficiency score of these 20 countries increased from 0.797 in 2000 to a peak at 0.82 in 2006 and then continually dropped to 0.745 in 2012. The reason for this phenomenon is probably that the proportion of the amount of CO_2_ emissions in these 20 countries in the total CO_2_ emissions of the world during 2000–2006 increased faster than during 2007–2012 while their GDP share of the total world decreased. The development of GDP growth and CO_2_ abatement in 2007–2012 is more unbalanced than that in 2000–2012.

Moreover, the average Efficiency scores of both the 10 Annex I countries and the 10 Non-Annex I countries show a continuous decrease during 2006–2012. Specifically, the average Efficiency score of the Non-Annex I countries continually dropped from the peak 0.734 in 2006 to 0.597 in 2012 while the average Efficiency scores of Annex I countries were around 0.89 over the whole period. However, the 10 Annex I countries saw a better Efficiency performance than the 10 Non-Annex I countries. Their average Efficiency scores during the period are 0.893 and 0.691, respectively. Specifically, Annex I countries saw a fluctuating but upward Efficiency trend, while Non-Annex I countries saw a relatively stable Efficiency performance during 2000–2002 and then reached the peak value of 0.734 in 2006, followed by a continuous decrease to 0.597 in 2012. The reason for the huge difference in the Efficiency performance between the Annex I countries and the Non-Annex I countries is perhaps that the Efficiency of countries in unfavorable circumstances is underestimated. Therefore, the effects of the external environment variables on inputs should be eliminated, and the Efficiency scores should be re-estimated.

In addition, being the largest CO_2_ emitter and the second largest economy in the world, China saw the smallest average score 0.151 of Efficiency in 2000–2012. Although the Chinese government announced its intention to develop a low carbon economy in 2007 and to reduce the intensity of CO_2_ emissions per unit of GDP in 2020 by 40%–45% compared to that in 2009, China’s Efficiency scores continued to drop from 0.154 in 2008 to 0.145 in 2011 and increased slightly to 0.147 in 2012.

### 3.2. Stage II: Using SFA to Quantify Environmental Effects

In stage II, SFA is employed to quantify the external environmental effects embedded in both the input and undesirable output slacks which can be obtained from the first stage of the DEA analysis. Therefore, there are four regression functions which take these three input slacks and one undesirable output slack as dependent variables in each regression function, respectively. All the independent variables in these four regression functions are the same, including GDP per capita, industry structure, government support, urbanization, and import and exports. The empirical results of SFA for each regression function are summarized in Table 4.

The results of Table 4 suggest that both industry structure and import and export have a positive relationship with the undesirable output CO_2_ emissions. This implies that the increase either in “the proportion of industry value added in GDP” or in “the proportion of import and export in GDP” contributes to the slacks increase of the undesirable output CO_2_ emissions, which would reduce the Efficiency.

Industrial production consumes a large amount of energy and brings about large-scale carbon emissions. The import and export also influence the carbon emissions positively, because they are possibly seldom related to the energy saving and emission reduction technology. Consequently, it is of great necessity to encourage the import and export of the energy saving and emission reduction technology.

Besides, the coefficient between industrial value-added and labors is positive, and that between industrial value-added and capital stock and energy consumptions is negative. This result suggests that a higher value of industrial value-added offers a favorable environment for reducing the slacks of capital stock and that of energy consumptions, but an unfavorable environment for reducing the slacks of labors.

Moreover, both government support and industry structure show a negative relationship with the slacks of energy consumptions and capital stock, but positive relationship with the slacks of labors. This implies that an increase in the proportion of general government final consumption expenditure in GDP (%) and the proportion of industry value added in GDP contributes to reducing these slacks of energy consumptions and capital stock.

In addition, urbanization shows a positive relationship with all three inputs, but negative relationship with the undesirable output CO_2_ emissions. The relationship between GDP per capita and CO_2_ emissions is weak and not significant.

### 3.3. Stage III: Re-Estimate Efficiency Using Adjusted Data

At this stage, the estimated coefficients from the regressions are used to predict the total input slack for each input and country based on its environmental variables. These predicted values are utilized to adjust the primary input data for each country according to the difference between the maximum predicted slack of the sample and the predicted slack pertaining to that country.

Then, we use the adjusted inputs and undesirable output which eliminates the environmental variable and random factor in Equation (2) and the original desirable output variable to re-estimate the Efficiency of the 20 countries. Therefore, the Efficiency purged of the effects of the external environmental factor and statistical noise can be obtained (shown in Table 5).

As is shown in Table 5, nine countries have the Efficiency value of “1” in 2000–2012. However, these countries are different from the nine countries which have the Efficiency of “1” in Table 3. Comparing between the two stages, the United States, Japan, United Kingdom, Australia, France in Annex I and Thailand in Non-Annex I remain efficient. The changes are as follows: in Annex I countries, Italy becomes inefficient while Spain shows efficiency; in Non-Annex I countries, Mexico and Iran are efficient but Saudi Arabia and South Africa are no longer efficient.

It is suggested that the Efficiency of Annex I countries mainly increased from 2000 to 2005, and then decreased from 2005 to 2012. By contrast, the Efficiencies of Non-Annex I countries was mainly reduced during the 12 years. The average value of the total 20 countries also shows a downward trend in that period, which indicates that the decline of undesirable output is insufficient compared with the growth of the desirable output.

The result implies the gap of the Efficiency performance between Annex I and Non-Annex I countries are not obvious. This conclusion is also supported by the evidence that the gap in the average score of Efficiency between Annex I countries and Non-Annex I countries is small, which are 0.993 and 0.941, respectively. The average score of the top 20 CO_2_ emitters Efficiency shows a downward trend during the period, which decreased from 0.98 in 2000 to 0.95 in 2012. In stage 3, the average Efficiency of Non-Annex I countries is lower than that of Annex I countries, which confirms that external environmental variables show significant impact on Efficiency.

Moreover, as the largest economy and the largest CO_2_ emissions per capita in the world, the United States stood at the Efficiency frontier during the whole period from 2000 to 2012. The performance of Efficiency in four countries (Japan, Mexico, France and Spain) became inefficient to efficient in the period, while the score of Efficiency in Korea and Canada continued to decrease.

### 3.4. Comparison of Results between Stage I and Stage III

In order to compare the different performances, Table 6 displays the descriptive statistics of Efficiency calculated in the first stage which contains the environmental effects and Efficiency obtained from the third stage which eliminates the external influences. In general, after controlling the external environment effects, the Efficiency scores in all countries were increased which results in the increase of the average score of Efficiency, the maximum score of Efficiency, and the minimum score of Efficiency in the overall countries, Annex I countries, and Non-Annex I countries. In the third stage, the average Efficiency scores of the 10 Annex I countries, the 10 Non-Annex I countries, and all the 20 countries are close to 1 which are respectively 0.993, 0.941 and 0.967, while their average Efficiency scores in the first stage are respectively 0.893, 0.691 and 0.792. However, the tendency of Efficiency scores overtimes in the third stage is similar to that in the first stage, which shows a continuous decrease. The increase of the Efficiency scores illustrates that under the condition of controlling the environmental variables, the benefit to countries operating under favorable circumstances was greater than the penalty suffered by countries operating under unfavorable circumstances.

Moreover, the number of efficient countries decreased, which indicates that the countries operating under favorable circumstances were judged to be efficient improperly due to the comparison with those operating in unfavorable circumstances.

Although the Efficiency of all the 20 countries increased while eliminating the influence of external environment, the Non-Annex I countries obtained a more obvious promotion. This suggests that the external environment has a more significant impact on Non-Annex I countries. Specifically, most countries in Non-Annex I is developing countries in which the mass production and less developed economy give rise to the fast increase of carbon emission. Moreover, the adjustment of the external environmental factors in these countries can bring about the balance of economic development and the carbon emission more significantly.

In addition, the standard deviation of the Efficiency score in these countries also decreased, which implies that without controlling the external environment variables, the countries operating in favorable circumstances saw biased upward Efficiency score while the countries operating in unfavorable circumstances saw biased downward Efficiency score. The adjustment of the inputs in the second stage put all the countries in the same environment and removed the Efficiency scores biases above. As a result, the spread was reduced.

The Pearson correlation coefficients between the Efficiency scores of the first stage and that of the third stage in Annex I countries, Non-Annex I countries and all the 20 countries are −0.294, 0.859 and 0.874, respectively. The coefficient in both the Non-Annex I countries and all the 20 countries are significant at the level of 1%. The non-significant coefficient of the Annex I countries indicates that adjusting the inputs according to external environmental variables affecting Efficiency makes an obvious difference in Efficiency scores of the Annex I countries.

The Spearman rank correlation coefficients between the Efficiency scores of the first stage and the third stage in Annex I countries, Non-Annex I countries and all the 20 countries are −0.429, 0.764 and 0.709, respectively. Again, the coefficient in both the Non-Annex I countries and all the 20 countries are at the level of 1% and 5%, respectively. The non-significant coefficient of the Annex I countries indicates that controlling the external environment affects Efficiency rankings among the countries.

Both the Pearson and Spearman measures indicate that controlling the external environmental variables in the development of low carbon economy significantly affects the Efficiency scores of the top 20 CO_2_ emission countries, as well as their Efficiency rankings.

## 4. Conclusions

In order to prevent global warming, a low carbon economy is introduced as a new economic mode to promote carbon abatement without damaging economic growth. Therefore, improving the efficiency of low carbon economy is a critical policy issue which attracts attention all over the world. This paper employs a three-stage approach to evaluating the efficiency of low carbon economy development in the top 20 CO_2_ emitting countries mainly by controlling the country variations in external environmental influences.

The results from Stage I indicate that the performance of Efficiency in the top 20 CO_2_ emitting countries, especially the Non-Annex I countries, had worsened as their average Efficiency scores show a continuous decrease during 2006–2012.

The SFA results in Stage 2 reveal that the increase in both the proportion of general government final consumption expenditure in GDP (%) and that of industry value added in GDP (%) can reduce input slacks to improve Efficiency. Moreover, it also indicates that increasing the proportion of urban population in total (%) can reduce the undesirable output CO_2_ emissions to improve Efficiency.

As a result of eliminating the impacts of external environment variables in Stage 2, the average Efficiency scores of both the Annex I countries and the Non-Annex I countries in the third stage differ from those in Stage 1. However, the tendency of Efficiency scores in the first and third stage shows a continuous decrease. Moreover, the performance of Efficiency in Annex I countries is better than that in Non-Annex I countries in both Stage 1 and Stage 3. However, after eliminating the impacts of external environmental variables, the gap of the average Efficiency score between Annex I and Non-Annex I countries in the third stage is smaller than that in the first stage. This result indicates that the external environment variables show greater influence on Non-Annex I countries than Annex I countries. In addition, as the largest economy and the second largest CO_2_ emitter in the world, the United States shows an Efficiency performance of developing low carbon economy as its Efficiency score in both the first and the third stage remains at “1” throughout the period. However, China, the largest CO_2_ emitter and the second largest economy in the world, saw the smallest Efficiency score among the 20 countries. This implies that the Chinese government should pay more attention to promoting low carbon economy development.

Our findings have important policy implications. The downward tendency of Efficiency score in the majority of these 20 countries indicates that these countries should pay more attention to promoting low carbon economy development. Moreover, the lower Efficiency scores of the Non-Annex I countries indicates that more efforts should be made by these countries to improve the low carbon economy efficiency. Especially, it is of pivotal importance in China which has the smallest Efficiency score among these 20 countries while the amount of CO_2_ emissions is the largest in the world. However, after controlling the external environmental factors, the scores of Efficiency in all countries are greatly improved. The increase of Efficiency indicates that these external environment variables which reflect the economic features of a country indeed have an impact on the performance of low carbon economy development. Additionally, the impact is larger for the Non-Annex I countries because the gap of the average Efficiency scores between Non-Annex I countries and Annex I countries is smaller in the third stage than that in the first stage. Therefore, the economic features of a country, including the industry structure, the process of urbanization, etc. should be taken into account when negotiating the responsibility of promoting CO_2_ reductions and developing low carbon economies among different countries. Most importantly, in light of the significant effect of the external environment on the Efficiency, the developed countries (mostly in Annex I) should help the developing countries (mostly in Non-Annex I) to reduce carbon emission by opening or expanding the trade, widening import and export, etc. It is of pivotal importance to encourage the import and export of the energy-saving and emission reduction technology, which would reduce the adverse impact of the import and export trade on carbon emission reduction.

## Figures and Tables

**Table 1:** Summary statistics of input and output factors by region (2000–2012).

Countries	Inputs						Output		Undesirable Output	
	E (100 Mtce)		K (1010)		L (1 Million Persons)		GDP (1010)		CO_2_ (100 Million Tonnes)	
	Mean	SD	Mean	SD	Mean	SD	Mean	SD	Mean	SD
*Annex I countries*										
United States	2296.7	51.8	276.4	31.1	153.6	4.2	1330.7	198.5	6255.8	232.9
Russia	659.1	26.1	21.3	14.4	75	1.8	102.4	62.5	1626.3	54.3
Japan	510.9	19.5	106.3	10.4	66.6	0.7	479.6	63.8	1358.7	51.4
Germany	327.2	11.7	51.9	10.5	41.3	0.8	286	64.1	866.9	42.5
Canada	316.6	10.6	26.3	9.6	17.9	1	122.2	39.4	617.5	17.9
United Kingdom	218.7	9.4	35.7	7.5	31	1.1	219.1	44.5	577	30.1
Italy	176.8	7	36	8.2	24.3	0.5	178.1	41.6	467	29.3
Australia	119.5	6.8	22	10.8	10.8	0.8	81.6	38	382.9	20.2
France	255.6	6.8	42.8	12.3	28.8	1	217.9	54.8	417.7	19.2
Spain	146.6	8.5	29.5	9.8	21.4	2.1	113.9	35	363.6	27.5
*Non-Annex I countries*										
China	1746.9	583.1	155.7	115.8	759.5	19.6	361.6	238.5	6006.7	1942.5
India	406.7	94.1	32	17.3	453.7	23.9	106.7	51.4	1310.2	301.6
Korea	227.1	26.2	23.8	5.8	24.2	0.9	83.5	22	631.1	75.9
Saudi Arabia	163.5	34	8.4	4.6	8.3	1.4	38.5	18.1	454.5	94.2
Iran	182.6	37.5	7.3	4.2	23.5	2.2	27.2	15.6	483.7	93.4
Brazil	220.1	32.6	22.7	14.2	94.9	6.9	126.2	70.8	405.1	53.8
Mexico	162.3	15.8	19.7	4.7	46.2	4	91.8	17.5	432.6	38
Indonesia	125.7	19.7	12.1	9.3	107.8	6.7	42.9	25.2	377.7	67.4
South Africa	115.9	9.5	4.7	2.2	18	0.7	25	9.8	419.7	34.7
Thailand	89.1	16.8	5.7	2.5	37.4	1.6	22	8.8	255.3	47.1

E: Energy consumption; K: Capital stock; L: Labor force; GDP: Gross Domestic Product; CO_2_: Carbon dioxide; SD: Standard Deviation.

**Table 2:** Descriptive statistics of external environmental variables.

Variables	Definition	Mean	SD
GDP per capita	The amount of GDP divided by midyear population (US$)	15,182.08	13,788.06
Government support	The proportion of general government final consumption expenditure in GDP (%)	16.99	4.24
Industry structure	The proportion of industry value added in GDP (%)	33.49	9.66
Import and exports	The proportion of exports and imports in GDP (%)	48.82	22.07
Urbanization rate	The proportion of urban population in total (%)	69.53	17.40

Note: all data are obtained from “World Bank Data”. Gross Domestic Product (GDP) per capita, Government support, Industry structure, Import and exports, and Urbanization refers to the indicators of GDP per capita (current US$), General government final consumption expenditure (% of GDP), Industry value added (% of GDP), Merchandise trade (% of GDP), and Urban population (% of total) in World Bank Data, respectively.

**Table 3:** The scores of Efficiency of the stage 1 in years 2000–2012.

DMU	2000	2001	2002	2003	2004	2005	2006	2007	2008	2009	2010	2011	2012
*Annex I countries*													
United States	1.000	1.000	1.000	1.000	1.000	1.000	1.000	1.000	1.000	1.000	1.000	1.000	1.000
Russia	0.330	0.304	0.316	0.315	0.325	0.340	0.343	0.329	0.323	0.311	0.303	0.293	0.292
Japan	1.000	1.000	1.000	1.000	1.000	1.000	1.000	1.000	1.000	1.000	1.000	1.000	1.000
Germany	0.757	0.789	0.884	1.000	1.000	1.000	1.000	1.000	1.000	1.000	1.000	0.909	0.954
Canada	0.709	0.714	0.692	0.692	0.683	0.686	0.711	0.687	0.693	0.716	0.709	0.691	0.692
United Kingdom	1.000	1.000	1.000	1.000	1.000	1.000	1.000	1.000	1.000	1.000	1.000	1.000	1.000
Italy	1.000	1.000	1.000	1.000	1.000	1.000	1.000	1.000	1.000	1.000	1.000	1.000	1.000
Australia	1.000	1.000	1.000	1.000	1.000	1.000	1.000	1.000	1.000	1.000	1.000	1.000	1.000
France	1.000	1.000	1.000	1.000	1.000	1.000	1.000	1.000	1.000	1.000	1.000	1.000	1.000
Spain	1.000	1.000	1.000	1.000	0.871	0.829	1.000	0.845	1.000	1.000	1.000	1.000	1.000
*Mean*	0.880	0.881	0.889	0.901	0.888	0.885	0.905	0.886	0.902	0.903	0.901	0.889	0.894
*Non-Annex I countries*													
China	0.175	0.175	0.166	0.147	0.137	0.137	0.139	0.149	0.154	0.145	0.145	0.145	0.147
India	0.306	0.291	0.297	0.290	0.263	0.255	0.248	0.238	0.232	0.214	0.214	0.205	0.204
Korea	0.560	0.551	0.557	0.545	0.537	0.557	0.591	0.570	0.549	0.545	0.525	0.517	0.539
Saudi Arabia	1.000	1.000	1.000	1.000	1.000	1.000	1.000	1.000	1.000	1.000	1.000	1.000	1.000
Iran	0.653	0.608	0.582	0.581	1.000	1.000	1.000	1.000	1.000	1.000	1.000	0.558	0.536
Brazil	1.000	1.000	1.000	1.000	1.000	1.000	1.000	1.000	0.598	0.592	0.562	0.532	0.524
Mexico	0.823	0.859	0.862	0.757	0.730	0.714	0.712	0.705	0.702	0.668	0.686	0.637	0.592
Indonesia	0.624	0.646	0.662	0.655	0.644	0.660	0.650	0.613	0.624	0.515	0.485	0.441	0.429
South Africa	1.000	1.000	1.000	1.000	1.000	1.000	1.000	1.000	1.000	1.000	1.000	1.000	1.000
Thailand	1.000	1.000	1.000	1.000	1.000	1.000	1.000	1.000	1.000	1.000	1.000	1.000	1.000
*Mean*	0.714	0.713	0.713	0.697	0.731	0.732	0.734	0.727	0.686	0.668	0.662	0.604	0.597
*Mean of the 20 countries*	0.797	0.797	0.801	0.799	0.809	0.809	0.820	0.807	0.794	0.785	0.781	0.746	0.745

DMU: Decision Making Unit.

**Table 4:** The results of Stochastic Frontier Analysis (SFA).

Variables	Slacks of			
	E	K	L	CO_2_
Constant	689.12 ***	26.91 ***	632.96 ***	2462.35 ***
Gross Domestic Product (GDP) per capita	−0.00078	−0.000042 ***	0.00003 ***	−0.0019
Government support	−44.08 ***	−1.95 ***	0.93 ***	−0.85
Industry structure	−9.54 ***	−0.11 ***	0.90 **	12.49 ***
Import and exports	−1.16	−0.08 ***	0.21 **	2.00 **
Urbanization	14.95 ***	0.40 ***	0.51 ***	−16.47 ***
Sigma-squared	237,647.09	433.34	39,319.84	2,796,319.9
Gamma	0.95	0.83	1.00	0.92
Log-likelihood function	−1662.93	−982.06	−1162.11	−2018.07
Likelihood Ratio (LR) test of the one-sided error	401.32	170.58	922.91	332.01

E: Energy consumption; K: Capital stock; L: Labor force; CO_2_: Carbon dioxide emissions; *** Significance at the 1% level. ** Significance at the 5% level.

**Table 5:** The scores of Efficiency of the stage 3 in years 2000–2012.

DMU	2000	2001	2002	2003	2004	2005	2006	2007	2008	2009	2010	2011	2012
*Annex I countries*													
United States	1.000	1.000	1.000	1.000	1.000	1.000	1.000	1.000	1.000	1.000	1.000	1.000	1.000
Russia	1.000	1.000	1.000	1.000	1.000	1.000	1.000	0.954	0.907	0.876	0.909	0.785	0.839
Japan	1.000	1.000	1.000	1.000	1.000	1.000	1.000	1.000	1.000	1.000	1.000	1.000	1.000
Germany	0.993	0.994	0.993	1.000	1.000	1.000	1.000	1.000	1.000	1.000	1.000	1.000	1.000
Canada	1.000	1.000	1.000	1.000	0.999	1.000	1.000	1.000	0.984	0.971	0.981	0.981	0.978
United Kingdom	1.000	1.000	1.000	1.000	1.000	1.000	1.000	1.000	1.000	1.000	1.000	1.000	1.000
Italy	1.000	1.000	1.000	1.000	1.000	0.999	0.987	0.993	1.000	1.000	1.000	1.000	1.000
Australia	1.000	1.000	1.000	1.000	1.000	1.000	1.000	1.000	1.000	1.000	1.000	1.000	1.000
France	1.000	1.000	1.000	1.000	1.000	1.000	1.000	1.000	1.000	1.000	1.000	1.000	1.000
Spain	1.000	1.000	1.000	1.000	1.000	1.000	1.000	1.000	1.000	1.000	1.000	1.000	1.000
*Mean*	0.999	0.999	0.999	1.000	1.000	1.000	0.999	0.995	0.989	0.985	0.989	0.977	0.982
*Non-Annex I countries*													
China	0.660	0.672	0.661	0.628	0.600	0.604	0.614	0.629	0.627	0.584	0.572	0.572	0.577
India	0.954	0.956	0.904	0.927	0.882	0.887	0.866	0.862	0.811	0.826	0.832	0.801	0.815
Korea	0.995	0.990	1.000	0.986	1.000	1.000	0.999	1.000	1.000	1.000	1.000	1.000	1.000
Saudi Arabia	1.000	0.999	1.000	1.000	1.000	1.000	1.000	1.000	1.000	1.000	1.000	1.000	1.000
Iran	1.000	1.000	1.000	1.000	1.000	1.000	1.000	1.000	1.000	1.000	1.000	1.000	1.000
Brazil	1.000	1.000	1.000	1.000	1.000	1.000	1.000	1.000	0.949	1.000	0.959	0.948	0.942
Mexico	1.000	1.000	1.000	1.000	1.000	1.000	1.000	1.000	1.000	1.000	1.000	1.000	1.000
Indonesia	1.000	1.000	1.000	1.000	1.000	1.000	1.000	1.000	1.000	1.000	1.000	0.798	0.844
South Africa	1.000	1.000	0.831	0.802	0.998	1.000	1.000	0.999	0.999	0.999	0.999	0.996	0.997
Thailand	1.000	1.000	1.000	1.000	1.000	1.000	1.000	1.000	1.000	1.000	1.000	1.000	1.000
*Mean*	0.961	0.962	0.939	0.934	0.948	0.949	0.948	0.949	0.938	0.941	0.936	0.911	0.917
*Mean of the 20 countries*	0.980	0.980	0.969	0.967	0.974	0.974	0.973	0.972	0.964	0.963	0.963	0.944	0.950

**Table 6:** The scores of Efficiency of the stage 1 in years 2000–2012.

	Stage 1	Stage 3
*All 20 countries*		
Mean of Efficiency scores	0.792	0.967
Std. Deviation of Efficiency scores	0.023	0.011
Maximum	0.745	0.944
Minimum	0.820	0.980
Pearson correlation coefficients		0.874 ***
Spearman rank correlation of Efficiency		0.709 ***
*Annex I countries*		
Mean of Efficiency scores	0.893	0.993
Std. Deviation of Efficiency scores	0.009	0.008
Maximum	0.880	0.977
Minimum	0.905	1.000
Pearson correlation coefficients		−0.294
Spearman rank correlation of Efficiency		−0.429
*Non-Annex I countries*		
Mean of Efficiency scores	0.691	0.941
Std. Deviation of Efficiency scores	0.046	0.015
Maximum	0.597	0.911
Minimum	0.734	0.962
Pearson correlation coefficients		0.859 ***
Spearman rank correlation of Efficiency		0.764 ***

*** Significance at the 1% level.
